# A Case of Anesthetic Management of Arnold-Chiari Malformation I: A Contest to Anesthesiologists

**DOI:** 10.7759/cureus.33848

**Published:** 2023-01-16

**Authors:** Vidur Mago, Vivek Chakole, Roshan Nisal, Roshan Umate

**Affiliations:** 1 Anesthesiology, Jawaharlal Nehru Medical College, Datta Meghe Institute of Higher Education and Research, Wardha, IND; 2 Research and Development, Jawaharlal Nehru Medical College, Datta Meghe Institute of Higher Education and Research, Wardha, IND

**Keywords:** scoliosis, video laryngoscopy, difficult laryngoscopy, hindbrain, malformation, chiari

## Abstract

Arnold-Chiari malformation is a very uncommon array of deformities in the posterior part of the cranium and hindbrain caused due to abnormal extension of the posterior brain into the spinal canal. Chiari malformation is further divided into subtypes 1, 2, and 3. The latter two are more common in pediatric forms and present at birth. The severity of symptoms depends upon the extent of herniation of the hindbrain due to herniation of the cerebellum through the foramen of the cranium. Also, there have been instances of absence of cerebellum. Multiple associated disorders like hydrocephalus due to increased intracranial pressure, then encephalocele, syrinx, or spinal deformity in the form of scoliosis have been presented in many cases. All these factors thus become a challenge to anesthesiologists for such patients. Hence evidence-based knowledge along with multidisciplinary, well-planned approach is required for its management.

## Introduction

The Arnold-Chiari malformation is characterized by herniation of the cerebellum, pons, and medulla oblongata leading to multiple symptoms, including intracranial and extracranial defects [1,2]. The etiology of the malformation is not exactly known, but varied theories have been proposed, including molecular, hydrodynamic, and mechanical mechanisms [3]. A smaller cranium leading to reduce volume causes downward displacement of the cerebellar tonsils out of the cranium through the foramen magnum in type I malformation. All these can be attributed to primary congenital hypoplasia or acquired morphological abnormalities like early closure of sutures or dysplasia, which has been seen in populations with chromosome 1 and 22 mutations leading to hereditary posterior fossa hypoplasia [4]. The cause of signs and symptoms is said to be due to direct compression of surrounding neural structures, including the spinal cord, and cerebrospinal flow obstruction leading to syrinx formation, which further causes pressure symptoms on the expansion of the cavity. Another factor attributing to the condition is the underdeveloped abnormal bone, leading to the prediction of space and herniation [3].

The patients usually present with headaches and neck pain, which increases when performing the Valsalva maneuver. Patients also present with ocular symptoms, hearing loss, vertigo, ataxia, sleep apnea, and fatigue. The severe stage of the disease also shows myelopathy leading to difficulty in deglutition and loss of pain and temperature in some patients. Major diagnostic tools for evaluation include magnetic resonance imaging (MRI) and routine blood investigations. The mainstay of treatment is surgery, where posterior fossa decompression is done, and a shunt is placed to drain the syrinx [4].

## Case presentation

**Figure 1:**
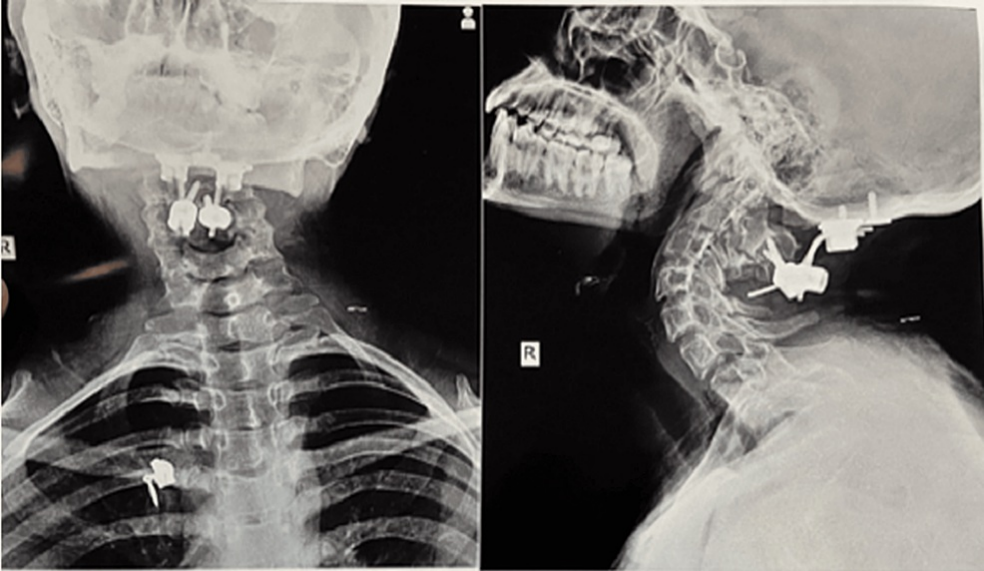
X-ray view depicting skeletal abnormality.

A 19-year-old female of 50 kg weight and 157 cm height came to our hospital with difficulty swallowing and speaking and right-side upper limb and lower limb weakness since birth for more than one month. The patient complained of difficulty swallowing more solids than liquids, speaking, and weakness on the right side. The patient had similar complaints in the past for which she was operated on for second cervical spine fixation along with fourth cervical spine laminectomy and syringostomy in 2015 under general anesthesia. Figure 1 shows skeletal abnormality in the patient.

On examination, the patient had an ataxic gait with a positive Romberg test. Bilateral pupils reactive to light, depressed gag reflex right>left side. A motor power assessment was done on the right upper limb, which was 4/5. The motor power of the right lower limb was 4/5, along with a decrease in tone, whereas power on the left side was 5/5 in the upper and lower limbs. The sensation was decreased by 50% below the level of the C6 vertebra, on the right side with loss of temperature sensation. The patient was planned for surgical intervention. In the preoperative period, nill by mouth (NBM) was confirmed, informed written consent for anesthesia was taken and she was shifted to the operating room. Monitors were attached and vitals were noted. Two wide-bore IV cannula 18G were secured, and preoxygenation was done with 100% oxygen. Premedication with Loxicard (2%) 2.5 mL (1 mg/kg), fentanyl (2 mcg/kg) 100 mcg IV, induction agent thiopentone (5 mg/kg) 250 mg IV, and vecuronium (0.1 mg/kg) 6 mg were given. The patient was intubated with the help of a video laryngoscope because of restricted neck extension, and reinforced tube 7.5 was inserted and fixed at the level of 21 cm from the lips. The patient was kept on ventilation mode with volume control mode, and tidal volume was set at 8 mL/kg, respiratory rate at 14 breaths per minute, and FiO_2_ at 50%.

**Figure 2:**
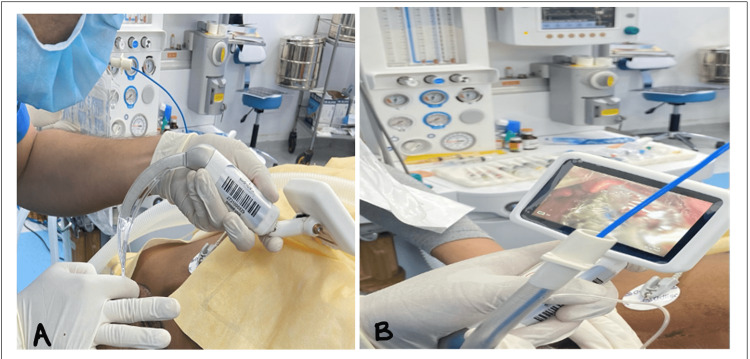
Video laryngoscope-assisted intubation (A and B).

Somatosensory evoked potential (SSEP) and bispectral index (BIS) monitor electrodes were connected. The patient was put into a prone position and proper positioning was checked. Anesthesia was maintained with propofol infusion at 30 mL/h (100 mcg/kg/min). A fentanyl bolus dose of 1 mcg/kg was given every hour. Strict BP monitoring was done. SSEP potential was noted at the time of tumor resection to avoid any neurological deficit during surgery. IV fluid was replaced according to requirement. Around 300 mL of blood loss and 400 mL of urine output were observed. The total duration of the surgical procedure was 7 h. After completion of the surgical closure, propofol infusion was stopped, and the patient was carefully shifted into a supine position. After the patient had signs of spontaneous breathing, thorough oral suctioning was done. The inhalational agent was stopped, and muscle relaxation was reversed with Myo Pyrolate (neostigmine with glycopyrrolate) 0.05 mg/kg. After the patient had spontaneous eye opening and followed commands, the balloon cuff of the endotracheal tube was deflated, and the patient was extubated. Post extubation patient was kept on 100% oxygen with a Hudson mask and observed for any neuro deficit. The patient was shifted to ICU and kept on oxygen support for observation. Figures 2A, 2B show video laryngoscope-assisted intubation.

## Discussion

The Arnold-Chiari malformation (ACM) type I comes with an array of challenges for the anesthesiologist including (1) skeletal deformity at the craniovertebral junction, (2) autonomic dysfunction, (3) abnormal response to neuromuscular blocking agents, and (4) increased intracranial pressure. The goal of anesthesia management for such patients has to be intensely addressing all aspects of the syndrome. In our case, we took thorough history and examination as the first step for anticipating challenges witnessed during the perioperative period. We looked for anatomical abnormalities and also assessed the respiratory, cardiovascular, and neurological functions of the patient. As autonomic dysfunction is well recognized in patients with ACM I, which can result in hemodynamic instability, hypotension, hypoxia, and hypercarbia in the intraoperative and postoperative period [5]. We prepared radial artery canulation for invasive blood pressure monitoring, and arterial blood gas analysis, for correction. Also, a warming blanket was put for temperature regulation.

ACM I patients present with skeletal abnormalities such as atlanto-axial or atlanto-occipital fission and scoliosis, which causes difficulty in intubation; similarly our patient also had scoliosis and limitation of neck extension for which patient required video laryngoscope-assisted intubation. SSEP and BIS monitoring were also placed for the measurement of sensitivity to neuromuscular blocking agents with train of four assessment and depth of anesthesia and to prevent overdosing on muscle relaxants. Another concern in our patient was raised intracranial pressure, which could increase due to multiple reasons, including laryngoscopy response, positioning, inadequate analgesia, and drugs used during anesthesia, hence proper consideration was done to overcome all the risk factors. Drugs such as ketamine were avoided for the same reason, also, emphasis was given to neuroprotective agents such as thiopentone for induction, and inhalational agents such as sevoflurane were preferred. Also, normocapnia was maintained throughout the surgery to avoid any rise in intracranial pressure, and peak airway pressure was maintained at 25 mmHg.

The reversal of neuromuscular blockade and activation was done cautiously after assuring the return of spontaneous respiratory function and the presence of airway protective reflexes. Sometimes patients of ACM I show absence of adequate breathing efforts or airway reflexes, the extubation in such patients should be delayed and weaning should be advised. In the postoperative period, patients can present with cardiac or respiratory failure because of autonomic dysfunction, hence our patient was kept in neurological intensive care unit for strict monitoring for at least 48 h postoperatively [6].

## Conclusions

The anesthetic management of patients with ACM undergoing surgical management is complex and requires preparedness and careful attention to avoid complications leading to morbidity and mortality. The key message of the study is that ACM I along with its complications, such as difficult airway, increased intracranial pressure, and autonomic dysfunction, makes it very challenging and requires well-structured and disciplined management by combined anesthesiology, neurology, and neurosurgery approach which emphasize on preoperative, intraoperative, and postoperative complications and its timely management.
